# A Case of Idiopathic Retroperitoneal Fibrosis Triggering Recurrent Gastrointestinal Bleeding

**DOI:** 10.7759/cureus.65039

**Published:** 2024-07-21

**Authors:** Zeyad Khalil, Rafael A Hanna, Soheir Maher

**Affiliations:** 1 College of Medicine, October 6 University, Cairo, EGY; 2 Emergency Medicine, Al-Fares Crystal Medical Complex, Makkah, SAU

**Keywords:** surgical resection, corticosteroid therapy, ischemic damage, mesenteric vessel encasement, gastrointestinal bleeding, idiopathic retroperitoneal fibrosis

## Abstract

Idiopathic retroperitoneal fibrosis (IRF) is a rare condition characterized by fibrous tissue proliferation in the retroperitoneal space, commonly affecting the ureters and other abdominal structures. This case report describes a previously undocumented presentation of IRF in a 52-year-old female, who presented with recurrent gastrointestinal bleeding and severe anemia over six months. Diagnostic workup included endoscopy, colonoscopy, abdominal computed tomography (CT), and biopsy, revealing fibrous encasement of the mesenteric vessels leading to ischemic damage and gastrointestinal bleeding. Treatment involved high-dose corticosteroids and surgical resection of the fibrotic tissue, which resulted in complete resolution of symptoms. The aim of this case report is to highlight this unique presentation of IRF, discuss the diagnostic challenges, and explore effective treatment strategies for managing this rare but significant complication.

## Introduction

Idiopathic retroperitoneal fibrosis (IRF) is a rare and enigmatic disorder characterized by the proliferation of fibrous tissue in the retroperitoneal space, often encasing abdominal structures, such as the ureters, aorta, and inferior vena cava [1]. The condition was first described by Ormond in 1948 and is sometimes referred to as Ormond’s disease [1]. The etiology of IRF remains largely unknown, although it is believed to involve an autoimmune or inflammatory process, possibly triggered by various factors, such as infections, medications, malignancies, and abdominal surgeries [2].

 It is hypothesized that the disease begins with an inflammatory process, leading to the activation of fibroblasts and the subsequent deposition of collagen and other extracellular matrix components in the retroperitoneal space [3]. This fibrous tissue proliferation results in the encasement and compression of retroperitoneal structures, which can lead to various clinical manifestations depending on the organs involved [3]. Chronic inflammation and fibrosis can cause significant morbidity due to organ obstruction and ischemia [3,4].

One key feature of IRF is the involvement of the ureters, leading to ureteral obstruction, hydronephrosis, and potential renal failure if left untreated [4]. Ureteral involvement can present as flank pain, hematuria, and signs of renal insufficiency. In more advanced cases, the fibrotic process may extend to other abdominal organs and structures, including the duodenum, pancreas, and mesenteric vessels, leading to diverse and often severe symptoms [5]. The mesenteric vessel involvement, although rare, can lead to ischemic damage and gastrointestinal complications.

Patients with IRF typically present with nonspecific symptoms, such as back pain, abdominal pain, weight loss, and malaise. These nonspecific symptoms often lead to a delayed diagnosis as they can be easily attributed to more common conditions [6]. In rare instances, the disease may involve the mesenteric vessels, leading to ischemic damage and gastrointestinal symptoms, as highlighted in this case report.

The diagnosis of IRF requires a combination of clinical, radiological, and histopathological findings. Imaging studies play a crucial role in diagnosing and assessing the extent of the disease. Computed tomography (CT) and magnetic resonance imaging (MRI) are the most commonly used modalities, providing detailed visualization of the fibrous tissue and its relationship with retroperitoneal structures [7]. These imaging techniques can reveal the characteristic appearance of a mass-like lesion surrounding the aorta and ureters, with varying degrees of enhancement. Advanced imaging modalities, such as positron emission tomography (PET) scans, may also be utilized to assess the metabolic activity of the fibrotic tissue and help differentiate IRF from malignant conditions [8].

Biopsy of the affected tissue is essential for confirming the diagnosis and excluding malignancies or other conditions that can mimic IRF. Histopathological examination typically shows dense fibrous tissue with varying degrees of inflammatory infiltrate, predominantly composed of lymphocytes and plasma cells [8]. Immunohistochemical staining may further aid in differentiating IRF from other fibrosing conditions, such as IgG4-related disease, which has overlapping histopathological features [8].

The management of IRF involves a combination of medical and surgical approaches. Corticosteroids are the cornerstone of medical treatment, effectively reducing inflammation and halting the progression of fibrosis in many cases [9]. The administration of high-dose corticosteroids can lead to significant symptomatic improvement and reduction in fibrotic tissue. Other immunosuppressive agents, such as azathioprine and mycophenolate mofetil, may be used as steroid-sparing agents or in refractory cases [10]. These agents help to maintain remission and reduce the adverse effects associated with long-term steroid use. In cases where the disease does not respond adequately to medical therapy or when there is significant organ obstruction, surgical intervention is often required to relieve obstruction and restore the function of affected organs. This can include ureterolysis, stent placement, or bypass procedures to alleviate ureteral obstruction and prevent renal failure [11].

In this unprecedented case, the patient presented with recurrent gastrointestinal bleeding as the primary symptom. The detailed diagnostic workup included endoscopy, colonoscopy, and abdominal CT, which revealed the fibrous encasement of the mesenteric vessels, leading to ischemic damage and gastrointestinal bleeding. The endoscopic findings were consistent with ischemic colitis, and the biopsy confirmed the presence of fibrous tissue encasing the mesenteric vessels. The patient was successfully treated with high-dose corticosteroids and surgical resection of the fibrotic tissue, resulting in complete resolution of symptoms.

The aim of this case report is to highlight this unique presentation of IRF, discuss the diagnostic challenges, and explore effective treatment strategies for managing this rare but significant complication. We hope to enhance the understanding and awareness of IRF and the importance of considering IRF in the differential diagnosis of unexplained gastrointestinal bleeding. 

## Case presentation

A 52-year-old female presented to the emergency department with her husband, complaining of recurrent episodes of gastrointestinal bleeding over the past six months. She reported intermittent melena and significant fatigue. The patient had been admitted to the hospital five times within the last six months for similar complaints and had received multiple blood transfusions due to severe anemia. She had no significant past medical history, no known allergies, and no family history of gastrointestinal or autoimmune diseases. Socially, she is a non-smoker and does not drink alcohol. On physical examination, the patient appeared pale and fatigued. Her vital signs were blood pressure of 110/70 mmHg, heart rate of 95 bpm, respiratory rate of 18 breaths per minute, and temperature of 36.8°C. Abdominal examination revealed mild tenderness in the epigastric region without any palpable masses or organomegaly. Rectal examination confirmed the presence of melena.

Laboratory findings (Table 1) included a hemoglobin level of 7.2 g/dL (normal: 12-15 g/dL), hematocrit of 22% (normal: 36-46%), white blood cell count of 8,500/mm³ (normal: 4,000-11,000/mm³), platelets of 220,000/mm³ (normal: 150,000-450,000/mm³), serum creatinine of 0.9 mg/dL (normal: 0.6-1.2 mg/dL), blood urea nitrogen (BUN) of 14 mg/dL (normal: 7-20 mg/dL), and liver function tests within normal limits. Diagnostic workup included endoscopy and colonoscopy, which revealed multiple areas of ischemic colitis with ulcerations but no active bleeding at the time of examination. An abdominal CT scan showed a mass-like lesion surrounding the aorta and extending to the mesenteric vessels, suggestive of retroperitoneal fibrosis. Further imaging with MRI confirmed the presence of a dense fibrous mass encasing the mesenteric vessels. A biopsy of the affected tissue showed dense fibrous tissue with lymphocytic infiltration, consistent with IRF. 

**Table 1:** Detailed laboratory findings over time

Test	January 1, 2023	March 15, 2023	June 10, 2023	August 5, 2023	September 1, 2023	October 15, 2023	December 1, 2023
Hemoglobin (g/dL)	11.0	8.5	7.2	6.8	7.8	11.5	13.0
Hematocrit (%)	34	28	22	21	24	35	40
White blood cell count (/mm³)	7,500	8,000	8,500	8,800	7,900	8,200	7,700
Platelets (/mm³)	230,000	220,000	220,000	215,000	225,000	220,000	215,000
Serum creatinine (mg/dL)	0.8	0.9	0.9	0.9	0.8	0.9	0.8
Blood urea nitrogen (BUN) (mg/dL)	12	13	14	14	13	14	13
Alanine transaminase (ALT) (U/L)	22	24	23	25	23	22	23
Aspartate aminotransferase (AST) (U/L)	18	20	19	21	20	19	20

The recurrent gastrointestinal bleeding in this patient was attributed to ischemic damage caused by the encasement of the mesenteric vessels by the fibrotic tissue. This rare manifestation of IRF led to compromised blood flow to the gastrointestinal tract, resulting in ischemic colitis and subsequent bleeding. A potential reason for this unusual presentation could be the extensive involvement of the mesenteric vessels by the fibrotic process, which is not commonly seen in IRF and suggests a more aggressive disease course in this patient.

On March 15, 2023, an endoscopy was performed to investigate the cause of the patient's recurrent gastrointestinal bleeding and persistent melena. Given her severe anemia and previous episodes of gastrointestinal bleeding, it was crucial to identify the source of bleeding and assess the extent of mucosal damage. The decision to perform an endoscopy was based on the need for a direct visualization of the gastrointestinal tract, which would allow for accurate diagnosis and targeted treatment.

**Figure 1:**
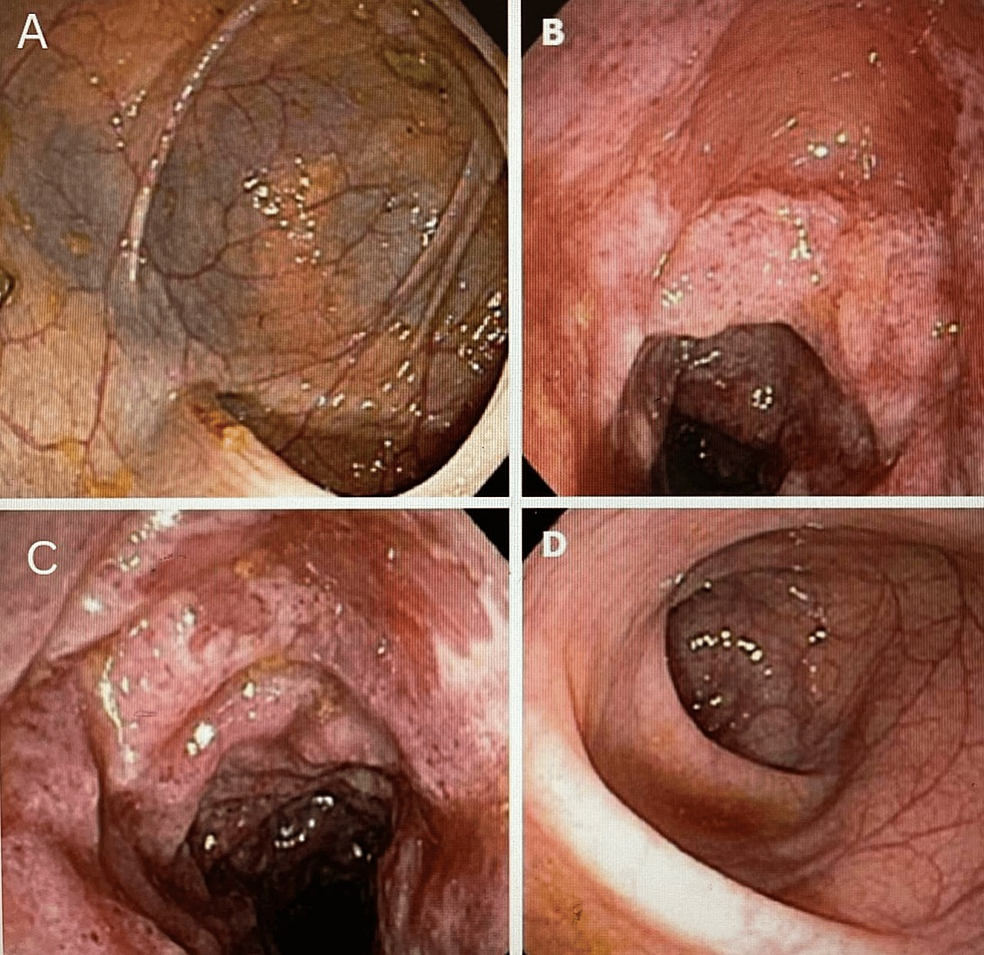
Endoscopic view showing ischemic colitis and ulcerations Image A shows the mucosal surface of the colon with notable pallor and a network of prominent blood vessels, indicative of ischemic injury. The mucosa appears thin and friable, with visible areas of submucosal hemorrhage and edema. There are also scattered yellowish exudates, suggesting early necrosis or fibrin deposition. Image B highlights a segment of the colon with significant erythema and ulceration. The mucosal surface is irregular, with patchy areas of ulceration that have undermined the normal architecture. This image clearly depicts the acute inflammatory response to ischemic injury, with evident hyperemia and mucosal breakdown. Image C demonstrates a more severe form of ischemic damage with deep ulcerations and necrotic tissue. The ulcers are surrounded by inflamed, erythematous mucosa, and there is evidence of hemorrhagic areas. This image underscores the extent of mucosal ischemia, leading to significant tissue destruction and potential for bleeding. Image D shows a relatively less affected area with only mild erythema and some mucosal edema. The vascular pattern is still visible, but there is less severe ulceration compared to the other images. This represents an area of early or mild ischemic injury, highlighting the variability of ischemic damage within the colon.

During the procedure, the patient was placed under conscious sedation to ensure comfort and cooperation. A flexible endoscope was carefully advanced through the patient's rectum and guided through the colon. The endoscope, equipped with a camera and light source, transmitted real-time images to a monitor, allowing for a detailed examination of the mucosal surface, as shown in Figure 1. As the endoscope was maneuvered through the colon, various segments were inspected for signs of inflammation, ulceration, and ischemic damage.

**Figure 2:**
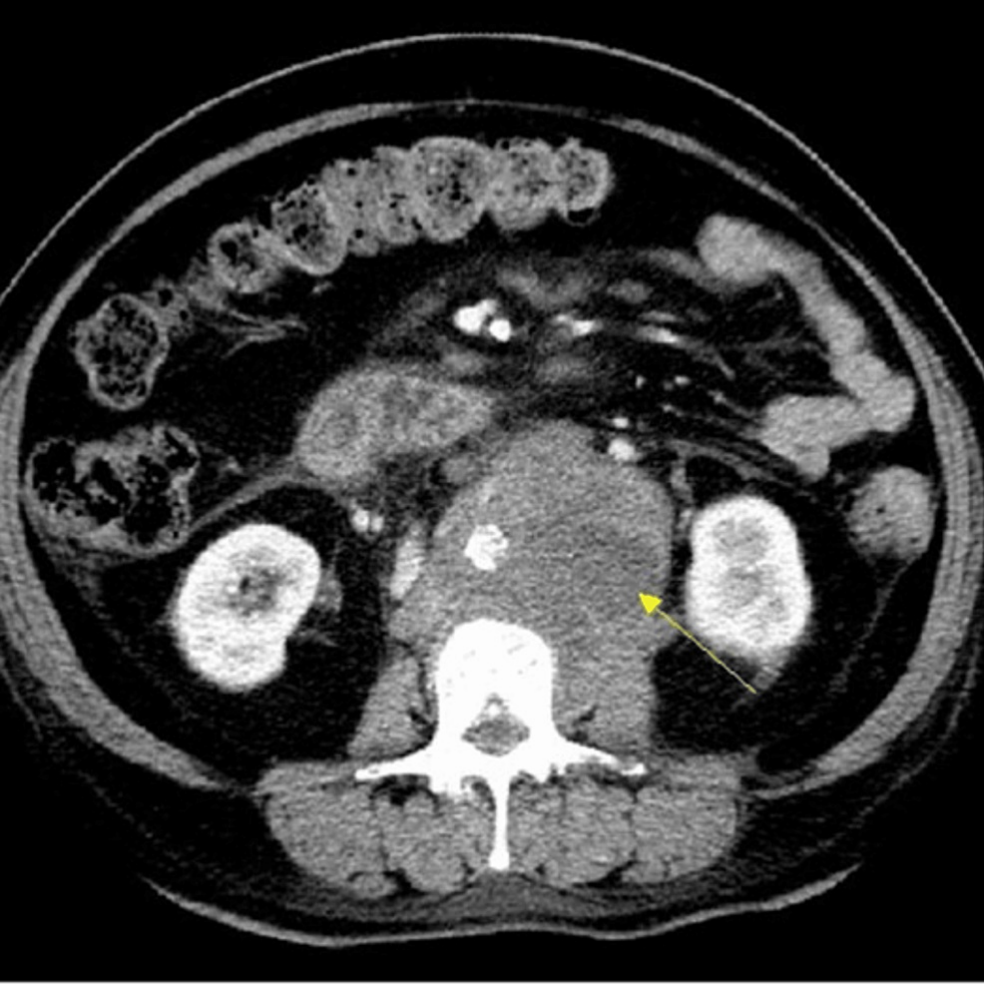
Abdominal CT showing multiple masses anterior to the aorta

The provided abdominal CT scan image (Figure 2), dated September 1, 2023, illustrates significant findings indicative of IRF. The scan reveals a dense, mass-like lesion in the retroperitoneal space, prominently encasing the aorta and extending to involve the mesenteric vessels, as indicated by the yellow arrow. This fibrotic mass exerts pressure on the surrounding structures, including the kidneys, which are visible on either side of the mass. The CT scan was performed to investigate the cause of the patient's recurrent gastrointestinal bleeding and severe anemia. The imaging findings confirmed the presence of extensive fibrous tissue proliferation, consistent with IRF. This condition led to compromised blood flow to the gastrointestinal tract, resulting in ischemic colitis and subsequent bleeding. 

The patient was started on high-dose corticosteroids (prednisone 60 mg daily) to reduce inflammation and fibrosis. Due to the severity of her symptoms and the risk of ongoing ischemic damage, surgical resection of the fibrotic tissue encasing the mesenteric vessels was performed. The surgical procedure involved careful dissection and removal of the fibrous tissue to restore adequate blood flow to the gastrointestinal tract. Postoperatively, the patient showed significant improvement in symptoms, with resolution of melena and stabilization of hemoglobin levels. Follow-up imaging showed no recurrence of the fibrotic mass, and the patient was maintained on a tapering dose of corticosteroids and monitored regularly for any signs of recurrence.

## Discussion

The development of IRF leading to recurrent gastrointestinal bleeding is a rare and complex phenomenon. Although the precise mechanisms behind IRF remain elusive, it is clear that this condition involves a robust inflammatory and fibrotic response within the retroperitoneal space. In the present case, the extensive fibrotic tissue encasement of the mesenteric vessels resulted in significant ischemic damage to the gastrointestinal tract, manifesting as recurrent episodes of melena and severe anemia. This underscores the multifaceted nature of IRF and its potential to affect various organ systems atypically.

The diagnostic approach to IRF requires a combination of advanced imaging techniques and histopathological confirmation. In this case, the initial identification of ischemic colitis through endoscopy and colonoscopy provided critical clues. These procedures revealed mucosal pallor, submucosal hemorrhage, and ulcerations, which further investigated potential underlying causes of ischemic damage [5]. The subsequent abdominal CT scan and MRI were instrumental in visualizing the extent of fibrous encasement and assessing the involvement of the mesenteric vessels. CT imaging, with its ability to provide detailed cross-sectional views, revealed a mass-like lesion in the retroperitoneal space. MRI further delineated the fibrous tissue characteristics and its relationship with surrounding structures, confirming the diagnosis of IRF [7].

The management of IRF often involves a multidisciplinary approach, incorporating both medical and surgical strategies. High-dose corticosteroids, such as prednisone at 60 mg daily, are the cornerstone of medical therapy. These agents work by suppressing the inflammatory response and halting the progression of fibrosis [9]. In many cases, corticosteroid therapy leads to significant symptomatic improvement and reduction in fibrous tissue. However, the adverse effects of long-term steroid use necessitate the consideration of steroid-sparing agents, such as azathioprine or mycophenolate mofetil, which can help maintain remission and minimize side effects [10].

Surgical intervention plays a crucial role in cases where fibrotic tissue causes significant obstruction or ischemia. In this patient, surgical resection of the fibrous tissue encasing the mesenteric vessels was essential to restore adequate blood flow to the gastrointestinal tract. The procedure involved meticulous dissection and removal of the fibrotic mass, alleviating the vascular compression and preventing further ischemic damage [11]. Postoperative follow-up with imaging ensured the absence of recurrence and monitored the effectiveness of ongoing medical therapy [11].

Overall, this case highlights the importance of considering IRF in the differential diagnosis of unexplained gastrointestinal bleeding and underscores the value of a comprehensive diagnostic and therapeutic approach. Advanced imaging techniques and tailored medical and surgical interventions are pivotal in managing this complex condition. The case also emphasizes the need for continued research into the underlying mechanisms and potential triggers of IRF, which could lead to improved diagnostic tools and therapeutic strategies in the future [2,3].

## Conclusions

This case report highlights the diagnostic challenges and complexities associated with IRF, particularly when it presents with atypical symptoms, such as recurrent gastrointestinal bleeding. The multifaceted nature of IRF, involving robust inflammatory and fibrotic responses, underscores the necessity of a comprehensive diagnostic approach that includes advanced imaging techniques and histopathological confirmation. The successful management of this patient through a combination of high-dose corticosteroids and surgical resection of the fibrotic tissue illustrates the importance of a multidisciplinary treatment strategy. This case emphasizes the need for heightened clinical awareness of IRF and its potential to affect various organ systems, even in the absence of classic symptoms. Future research should focus on elucidating the underlying mechanisms and potential triggers of IRF, aiming to improve diagnostic accuracy and therapeutic outcomes for this rare but significant condition.
